# Effects of seasonality and different rearing systems on the behavior and thermal comfort of sheep in the northern region of Brazil, eastern Amazon

**DOI:** 10.3389/fvets.2026.1789845

**Published:** 2026-05-07

**Authors:** Rubens Lima de Andrade, Raquel Silva de Holanda, Carlos Eduardo Lima Sousa, Thiago Nogueira da Silva, Luís Gustavo Paixão Vilela, Katarina Cardoso de Carvalho, Tatiane Silva Belo, Erica Silva de Oliveira, Wellen Karolyne Martins Ribeiro, Rafalel Ricardo Sampaio Rodrigues Bruxel, Bianca Sousa Campinas, Luíza Brito Barreto, Renata Umbelina Nogueira Campos da Silva, Jéssica Silva de Alencar, Lucietta Guerreiro Martorano, Cláudio Vieira de Araújo, Raimundo Nonato Colares Camargo-Júnior, Éder Bruno Rebelo da Silva, Vanessa Sousa Pinto, Italo Messias Ferreira de Souza, Welligton Conceição da Silva

**Affiliations:** 1 Department of Veterinary Medicine, University Center of the Amazon (UNAMA), Santarém, PA, Brazil; 2 Postgraduate Program in Animal Science, Institute of Agricultural and Environmental Sciences, Federal University of Mato Grosso (UFMT), Cuiabá, MT, Brazil; 3 Lutheran University Center of Santarém (CEULS/ULBRA), Santarém, PA, Brazil; 4 Embrapa Eastern Amazon, Santarém, PA, Brazil; 5 Federal Institute of Education, Science and Technology of Pará (IFPA), Santarém, PA, Brazil

**Keywords:** animal welfare, climate change, grazing, rumination, thermoregulation

## Abstract

Climate change causes physiological, metabolic, and behavioral changes that affect animal welfare (AW). Therefore, the objective of this study was to evaluate the effects of seasonality and different breeding systems on the behavior and thermal comfort of sheep in northern Brazil, in the eastern Amazon region. We used 20 Santa Inês sheep, non-castrated, non-pregnant, and non-lactating females. They were randomly divided into two systems: Traditional System (TS; *n* = 10), Silvopastoral System (SP; *n* = 10). The behavioral assessment was carried out from 6 a.m. to 6 a.m., with behavior recorded every 5 min, considering the morning (MH), afternoon (TD), night (NT), and dawn (MD) shifts, during the most and least rainy periods. The Temperature and Humidity Index (THI) was calculated. The data were evaluated at a 5% significance level using the software Statistical Analysis System (SAS). THI was higher during the afternoon shift, indicating severe stress (89%). There was a difference (*p* < 0.001) in the ingestive and idle behaviors of the sheep, influenced by seasonal periods, day shifts, and production systems evaluated. Grazing occurred with greater intensity in the less rainy period (64.81%) compared to the rainiest period (35.19%), being more pronounced during MD (42.40%) and MH (39.23%). Standing rumination was higher in the rainy season (56.71%) and in the NT (77.28%), while in the less rainy period, it occurred during MH (100%). It is concluded that rearing sheep in an SP favored a greater expression of AW behaviors, contributing to higher production efficiency.

## 1 Introduction

Sheep farming in Brazil is a prominent sector within national livestock production, contributing significantly to the economy and the development of agribusiness. According to data from the Brazilian Institute of Geography and Statistics (1), sheep farming is not only limited to the production of meat and milk but also includes wool production, generates employment, and stimulates economic activity in several regions of the country (2, 3). According to IBGE (1), Brazil currently has 21,862,326 sheep, corresponding to 65.17% of the total small ruminant population in the country, reflecting growth compared to previous years.

The breeding of small ruminants in Brazil presents significant challenges for producers. Sheep nutrition involves essential factors such as proteins, vitamins, energy, and minerals, which are critical for maintaining animal health and productive performance (4, 5). However, beyond nutritional management, several environmental and sanitary factors influence animal health and productivity. Parasitic diseases, inadequate management, sanitary deficiencies, heat stress, and climatic variations directly affect meat productivity and quality, as well as animal behavior (6).

In tropical regions such as the Amazon, high temperatures and elevated relative humidity intensify environmental challenges, requiring sheep breeds to adapt to these conditions (7). Heat stress occurs when animals are exposed to extreme thermal conditions that exceed their capacity to dissipate heat and maintain homeothermy. These thermal instabilities cause physiological, metabolic, and behavioral changes that affect animal wellbeing, reducing feed intake and weight gain (8). Such alterations compromise productive performance and negatively impact overall meat and wool production (9).

In the reproductive aspect, heat stress decreases conception rates, compromises semen quality in males, and alters the estrous cycle of females, reducing herd fertility (10). In addition, animals exposed to thermal stress show behavioral changes, including an increased search for shade, elevated respiratory rate, reduced physical activity, and greater susceptibility to diseases due to compromised immunity. In extreme situations, heat stress can lead to exhaustion, severe dehydration, and even death (11).

Although the impacts of heat stress on physiological and reproductive performance are documented, there is still limited information regarding the behavioral responses and thermal comfort of sheep raised under different production systems in humid tropical climates, particularly when seasonal variations are considered. Comparative evaluations between silvopastoral and traditional systems under these environmental conditions remain scarce, especially in the Eastern Amazon region.

Given this context, the present study was conducted to address the need to better understand the behavioral responses and thermal comfort of sheep raised in silvopastoral and traditional systems under humid tropical conditions. Therefore, the objective of this study was to evaluate the effects of seasonality and different breeding systems on the behavior and thermal comfort of sheep in northern Brazil, specifically in the Eastern Amazon region, thereby contributing to the development of management strategies aimed at improving animal welfare and production efficiency.

## 2 Materials and methods

### 2.1 Ethical aspects

The procedures adopted in this experiment followed the ethical standards of research, in accordance with current Brazilian laws, and were previously approved by the Ethics Committee on the Use of Animals (CEUA/UNAMA), under protocol number UNAMA-CEUA-2025-0002-08-WS.

### 2.2 Location and climate

The study was conducted on a rural sheep farm located in the municipality of Santarém, in the Tabocal community, Pará, Brazil. The area is situated at an altitude of approximately 51 m above sea level, which is characteristic of the lowland Amazon region. This relatively low elevation contributes to the predominance of high temperatures and elevated atmospheric humidity throughout the year.

The climate of the mesoregion is classified as hot and humid (Am4) according to the Köppen–Geiger climate classification system, which characterizes tropical monsoon climates with high temperatures throughout the year and a well-defined seasonal rainfall pattern (12). In this classification, the “A” denotes tropical climates with mean monthly temperatures above 18 °C, while “m” indicates a monsoon regime with a short dry season and high annual rainfall. The Am4 subtype, as described for the region, is characterized by total rainfall exceeding 60 mm in the wettest month and annual precipitation ranging between 1,900 and 2,100 mm. The average annual air temperature is 25.6 °C, and relative humidity ranges between 84 and 86% (13, 14).

The experiment was conducted during the least rainy period (July to December) and the rainiest period (January to June), allowing seasonal climatic variation to be incorporated into the analysis and enabling the evaluation of animal responses under contrasting environmental conditions.

### 2.3 Experimental animals, management, and characterization of the breeding system

We used 20 Santa Inês sheep, female, non-pregnant, non-lactating, non-castrated, and dark-coated (brownish-black), aged between 10 and 18 months, weighing 37.19 ± 6.17 kg, with a body condition score of 3.0 [scale of 1–5—(15)], clinically healthy, and kept under the same sanitary and dietary conditions. The sheep were randomly divided into two groups: Traditional System (TS; *n* = 10) and Silvopastoral System (SP; *n* = 10). The TS group was kept in a paddock without the shade of trees, with access to drinking water and mineral salt *ad libitum*. The SP group, in contrast, remained in paddocks of the same size and pasture, with access to 37% of shade provided by muruci trees (*Byrsonima crassifolia* (L.) Rich) and to drinking water and mineral salt *ad libitum*.

The total experimental area was 4,995 m^2^, cultivated with *Panicum maximum* cv. Mombasa, and divided into six paddocks of 27.5 m each, managed under rotational grazing with 20 days of occupation and 20 days of rest in three paddocks for each system, according to recommendations (16). An average annual stocking rate of 2 U.A. was considered. In pasture management, the nutrients necessary for both periods of the year were applied, corresponding to the less rainy period and the transition period, according to soil analysis.

### 2.4 Ethogram

In the evaluation of the animals, visual observation was used, considering the phenotypic characteristics of each animal, making it possible to identify them individually. For this, four trained observers were used, divided into pairs, being replaced every 2 h, avoiding fatigue, between one paddock and another, without influencing animal behavior. The animals were identified with earrings of different colors for each treatment and the use of different ribbons and coloring for each system, with the Nylon® ribbon being 20 mm thick, not interfering with the physiological behavior of the animals.

The animals were assessed once a month between 6 a.m. and 6 p.m. during the study period. Behavior assessment was performed every 5 min. For the analysis, four shifts were considered: morning (MH), afternoon (TD), night (NT), and dawn (MD). The behavioral terms used, as described in the ethogram, followed the existing basis of Santos et al. (17) and Junior et al. (18) (Table 1).

**Table 1:** Ethogram of sheep behavior predefined in the literature.

Behavior definition	
Standing	Leaning on your limbs, moving or standing still.
In bed	Animal with four legs flexed and its abdomen totally or partially in contact with the ground.
Grazing	The act of feeding on pasture in season.
Rumination	Chewing, swallowing, regurgitation, and rechewing of animals with the presence of the food bolus apparent in the cheek space, being performed standing or lying down.
Leisure	An inattentive look in any direction with no apparent purpose, lying down, or standing.
Walking	Animals moving without grazing inside the paddocks.

Adapted by Santos et al. (17) and Junior et al. (18).

The objective was to evaluate the variation between observers in relation to behavior patterns. Interobserver variation was assessed using the Kappa coefficient calculated in Microsoft Excel 2013 (Microsoft Corp., Redmond, WA, USA), and an interobserver reliability of 90% was identified.

### 2.5 Meteorological variables

The climatic variables evaluated were air temperature (AT, °C), relative humidity (RH%), wind speed (m/s), and dew point temperature (DPT °C). These variables were obtained through an intelligent portable weather station (IMPAC®; IP-232) and measured every 15 min during the experimental period.

#### 2.5.1 Temperature and humidity index (THI)

Throughout the experiment, simultaneous measurements of environmental and physiological variables were taken throughout the day. Based on the recorded values of environmental conditions, the THI, developed by Thom (19), was calculated using the following equation:

THI=DBT+0.36×DPT+41.5

where DBT is the dry bulb temperature (°C), and DPT is the dew point temperature (°C).

The THI is a measure that indicates the level of heat stress and is classified as follows: <72 indicates no stress; 72–78 indicates mild stress; 79–88 indicates moderate stress; and 89–98 indicates severe stress (20).

### 2.6 Statistical analysis

The experiment was conducted in a completely randomized design (DIC) in a split-plot structure (TS and SP), with the effect of the period characterizing the plot and the shifts defining the subplot. The statistical model is defined as:

Y_ijk=μ+α_i+α_i_((k))+β_j+(αβ)_ij+ε_ijk

where Y_ijk is the value of the response variable measured in period i, turn j and repetition k; μ is a general constant inherent in all observations in Y_ijk; α_i represents the effect of level i of the measurement factor; 〖α_i〗_((k)) represents the error inherent in the plot; β_j is the effect of the turn level j; (αβ)_ij is the effect of the interaction between the period and shift; and ε_ijk is the error associated with the measurement of Y_ijk in the experimental units k allocated in shift j and evaluated in period i. The hypotheses tested were H0: Σα_i = 0; Σβ_j = 0 and 〖αβ〗_ij = 0. The data were evaluated using the Statistical Analysis System (SAS) software, considering 5% significance.

## 3 Results

The data demonstrate differences in THI between shifts (MD, MH, NT, and TD) in both the rainy season and the less rainy season (*p* < 0.05). During the rainy season, THI was highest in the TD shift (81.01), followed by NT (77.14) and MH (76.94). In the less rainy season, THI values were higher overall, with the greatest values observed in TD (84.65), followed by MH (79.00) and NT (77.25) (Table 2).

**Table 2:** The temperature and humidity index (THI) in relation to periods and shifts.

Periods	Shift	THI
		Average/SD
More	Dawn	74.33 ± 1.38a
More	Morning	76.94 ± 2.44b
More	Night	77.14 ± 1.86b
More	Afternoon	81.01 ± 1.52c
General		78.36 ± 3.01
Less	Dawn	73.27 ± 1.02a
Less	Morning	79.00 ± 3.08b
Less	Night	77.25 ± 2.69b
Less	Afternoon	84.65 ± 1.27c
General		78.55 ± 4.64

Different letters (a, b) indicate statistical difference in the column (*p* < 0.05).

**Figure 1:**
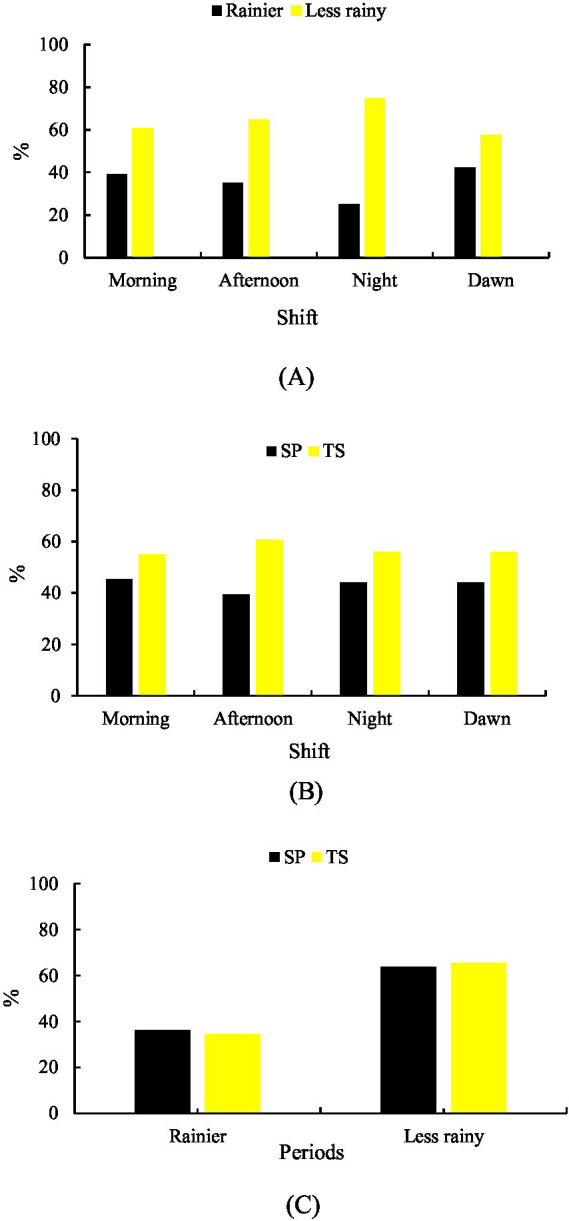
Grazing behavior. **(A)** Rainier and less rainy periods that are associated with shifts. **(B)** Shift-related systems. **(C)** Systems in relation to the period of the year.

A significant difference (*p* < 0.001) was observed between shifts in both the rainy season (35.19%) and the less rainy season (64.81%) for grazing behavior. During the rainy season, sheep grazed more frequently in the MD shift (42.40%), followed by MH (39.23%) and TD (35.02%). In contrast, during the less rainy season, grazing activity was highest in the NT shift (74.94%), followed by TD (64.98%) and MH (60.77%) (Figure 1A).

Differences were also observed between production systems (*p* < 0.001). In the silvopastoral system (SP), grazing was more frequent in the MH shift (45.23%), followed by MD (44.06%) and NT (44.04%). In the traditional system (TS), grazing activity was highest during the TD shift (60.53%), followed by MD (55.94%) and MH (54.77%) (Figure 1B).

Regarding seasonal comparison between systems, grazing was more frequent in SP during the rainy season (36.25%), whereas in the less rainy season, sheep were more active in TS (65.61%) (Figure 1C).

**Figure 2:**
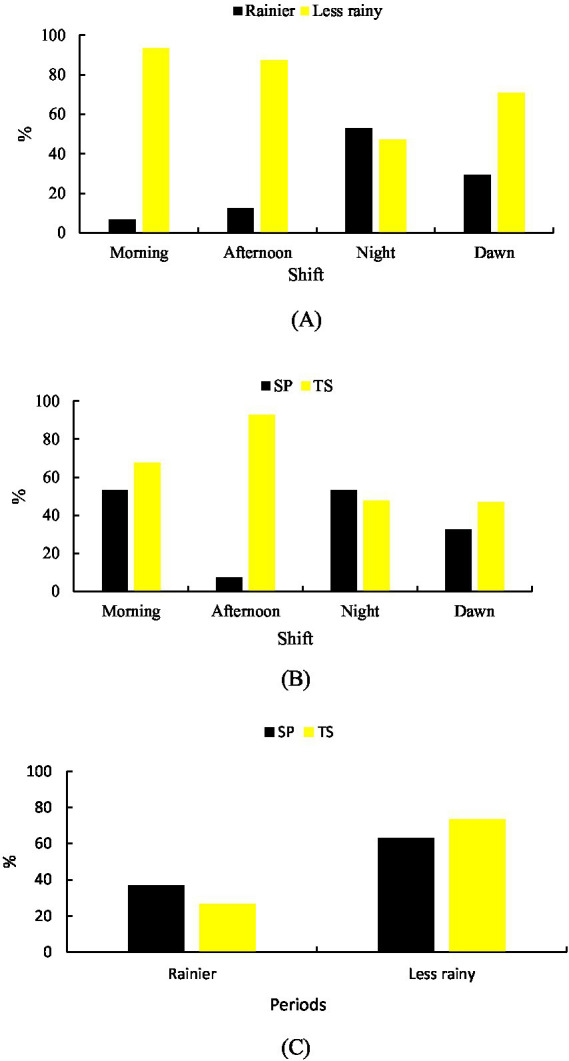
Leisurely behavior while standing. **(A)** Rainier and less rainy periods that are associated with shifts. **(B)** Shift-related systems. **(C)** Systems in relation to the period of the year.

Significant differences (*p* < 0.001) between shifts were observed for standing idleness in both the rainy season (30.26%) and the less rainy season (69.74%). During the rainy season, this behavior was most frequent in the NT shift (52.98%), followed by MD (29.34%) and TD (12.60%). In the less rainy season, standing idleness was highest in MH (93.30%), followed by TD (87.40%) and MD (70.66%) (Figure 2A).

Between systems, TS showed higher overall standing idleness (64.27%) compared to SP (35.73%) (*p* < 0.001). In SP, the highest frequencies were recorded in MD (53.28%) and NT (53.35%), whereas in TS, the highest frequency occurred in TD (92.72%) (Figure 2B).

Seasonally, standing idleness was more frequent in SP during the rainy season (36.94%), while in the less rainy season, it was higher in TS (73.45%) (Figure 2C).

**Figure 3:**
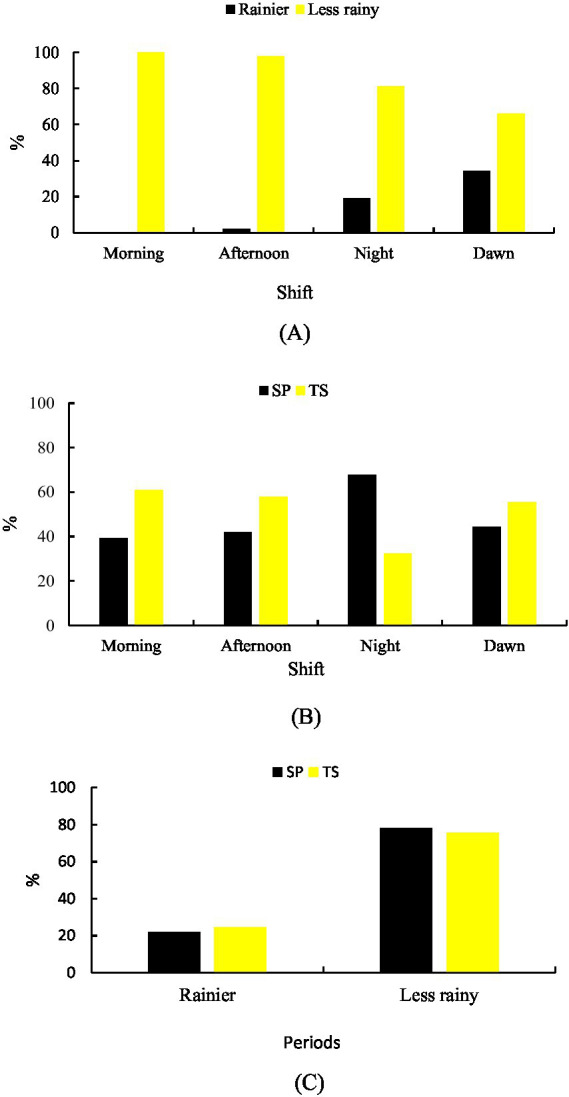
Idle behavior lying down. **(A)** Rainier and less rainy periods that are associated with shifts. **(B)** Shift-related systems. **(C)** Systems in relation to the period of the year.

Significant differences (*p* < 0.001) were observed between shifts for lying idleness in both seasons. During the rainy season (23.11%), the highest percentage occurred in MD (34.17%), followed by NT (18.94%). In the less rainy season (76.89%), the highest frequencies were observed in MH (100%), followed by TD (97.76%) and NT (81.06%) (Figure 3A).

Between systems, lying idleness was distributed similarly overall (SP 49.78%; TS 50.22%), but differed by shift (*p* < 0.001). In SP, it was most frequent in NT (67.64%), while in the TS, it predominated in MH (60.83%) (Figure 3B).

Seasonally, greater intensity of lying idleness was observed in SP during the rainy season (24.38%) and in TS during the less rainy season (78.16%) (Figure 3C).

**Figure 4:**
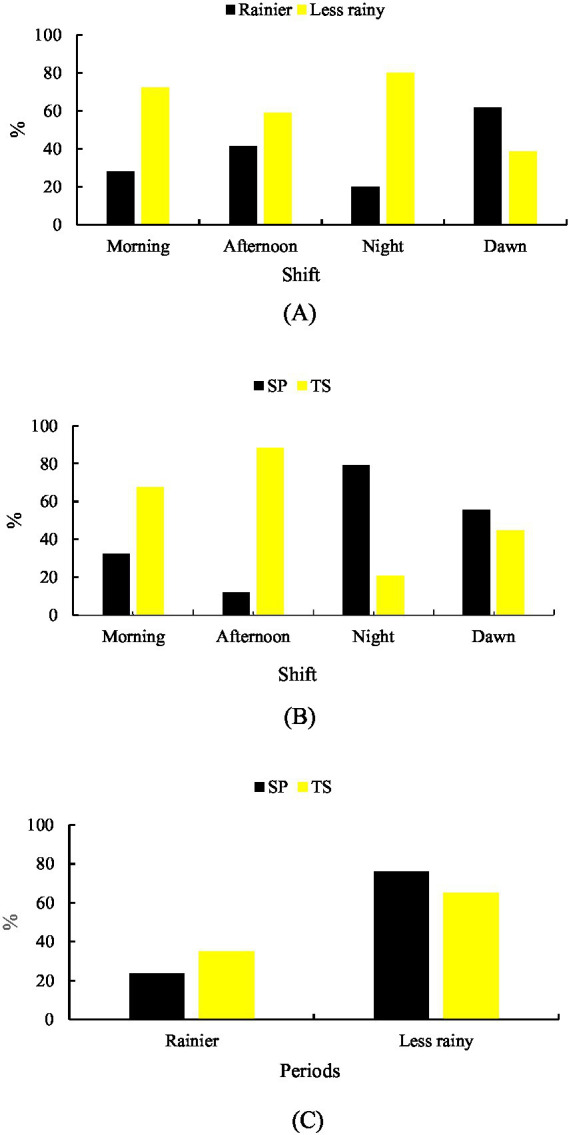
Walking behavior. **(A)** Rainier and less rainy periods that are associated with shifts. **(B)** Shift-related systems. **(C)** Systems in relation to the period of the year.

Walking behavior differed significantly (*p* < 0.001) between shifts in both the rainy season (29.82%) and the less rainy season (70.18%). In the rainy season, walking predominated in MD (61.54%), followed by TD (41.23%). In the less rainy season, the highest walking activity occurred in NT (79.95%), followed by MH (72.11%) (Figure 4A).

System comparison showed greater walking activity in TS overall (53.39%) than SP (46.61%) (*p* < 0.001). In SP, walking was highest during NT (79.20%), whereas in TS, it was highest during TD (88.16%) (Figure 4B).

Seasonally, walking was more frequent in TS during the rainy season (35.05%) and in SP during the less rainy season (76.18%) (Figure 4C).

**Figure 5:**
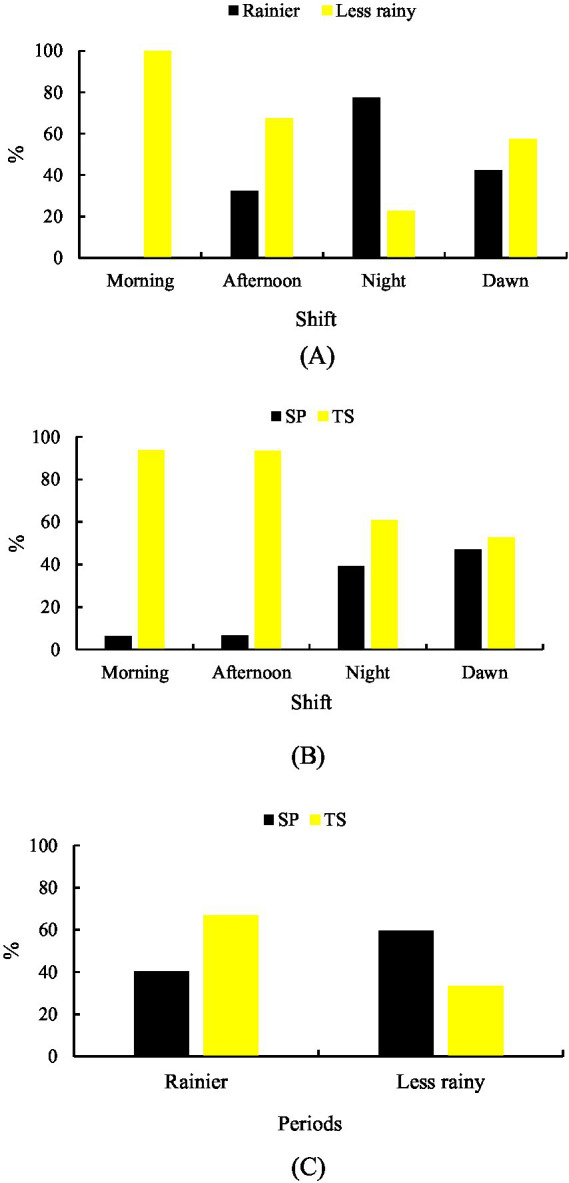
Behavior ruminating standing up. **(A)** Rainier and less rainy periods associated with shifts. **(B)** Shift-related systems. **(C)** Systems in relation to the period of the year.

Significant differences (*p* < 0.001) were observed between seasons for rumination while standing. During the rainy season (56.71%), the highest frequency occurred in NT (77.28%), whereas in the less rainy season (43.29%), it predominated in MH (100%) (Figure 5A).

SP accounted for 37.92% of total rumination standing, with a higher frequency in MD (47.23%). In TS (62.08%), the highest frequency was observed in MH (93.62%) (Figure 5B).

Seasonally, rumination while standing was higher in TS during the rainy season (66.69%) and higher in SP during the less rainy season (59.64%) (Figure 5C).

**Figure 6:**
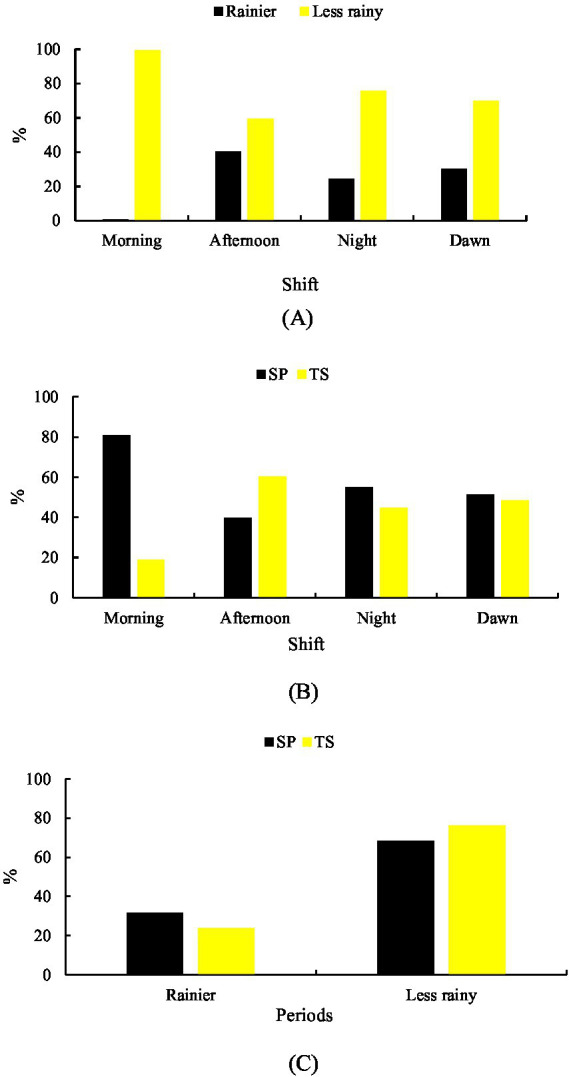
Behavior ruminating lying down. **(A)** Rainier and less rainy periods that are associated with shifts. **(B)** Shift-related systems. **(C)** Systems in relation to the period of the year.

Significant differences (*p* < 0.001) were also observed for rumination lying down between seasons. During the rainy season (27.99%), this behavior was highest in TD (40.51%). In the less rainy season (72.01%), it was highest in MH (99.35%) (Figure 6A).

In SP, rumination lying down was most frequent in MH (81.05%), whereas in TS, it predominated in TD (60.22%) (Figure 6B).

Seasonally, this behavior was more frequent in SP during the rainy season (31.58%) and in TS during the less rainy season (76.05%) (Figure 6C).

## 4 Discussion

The present results demonstrate that both climatic seasonality and the rearing system directly modulated sheep behavioral patterns, confirming that environmental pressure in humid tropical conditions significantly alters activity distribution throughout the day. The higher grazing frequency observed during the less rainy season indicates an adaptive response to seasonal forage dynamics. Reduced pasture availability and quality likely required greater foraging effort to meet nutritional demands, which aligns with the interpretation proposed by Santos et al. (21). However, unlike generalized assumptions that grazing simply increases under nutritional restriction, our data show that this increase was temporally redistributed toward cooler shifts (NT, TD, and MH), reinforcing that thermal load, not only forage scarcity, shapes behavioral expression (22).

The comparison between production systems further clarifies this interaction. In the traditional system (TS), grazing concentration during cooler periods suggests that the absence of shade restricts daytime activity due to thermal discomfort, corroborating the mechanisms described by Masters et al. (23) and Sejian et al. (24). In contrast, the silvopastoral system (SP) allowed greater temporal flexibility in grazing behavior, consistent with its moderated THI values. This supports the microclimatic buffering effect attributed to tree cover (25, 26). However, our findings also indicate that shade did not completely eliminate thermal constraints, particularly during TD, suggesting that silvopastoral systems mitigate, but do not fully neutralize, heat stress under high solar radiation (27, 28).

Standing idleness increased during the less rainy season, particularly in hotter shifts, supporting its role as a thermoregulatory strategy. Standing idleness enhances convective and radiative heat dissipation, as described by Shephard et al. (29) and Ayoola et al. (30). Interestingly, higher standing idleness in SP during TD suggests that even in buffered environments, peak radiation periods still impose physiological challenges (15, 31). This finding nuances the assumption that SP uniformly improves comfort and highlights that mitigation effectiveness depends on diurnal thermal peaks.

Lying idleness patterns revealed seasonal soil effects. The greater frequency during the less rainy season suggests that lower soil moisture increases suitability for recumbency, whereas saturated soils in the rainy season likely discouraged this behavior (32). Moreover, higher lying idleness in SP during NT and MD reinforces the role of stable microclimates in promoting rest under thermally favorable conditions (33, 34). These findings are consistent with Wang et al. (35) and Tüfekci et al. (36), who associate environmental buffering with improved welfare indicators, although our data emphasize that this benefit is seasonally modulated.

Walking behavior also reflected adaptive redistribution. Increased displacement during the less rainy season supports the hypothesis of greater spatial effort to locate forage under heterogeneous pasture distribution (23, 37, 38). However, the shift-specific differences reveal that locomotion is not solely nutritional in origin; it also serves thermoregulatory purposes. Greater movement during cooler shifts in both systems aligns with previous findings that locomotion decreases under high heat load (39–43). The higher walking activity in TS during TD likely reflects increased resource-seeking behavior in the absence of environmental protection (44, 45), whereas in SP, increased movement during the less rainy season may indicate environmental enrichment and greater spatial use (46–50). This contrast demonstrates that identical behaviors may arise from distinct environmental drivers.

Rumination patterns further support behavioral compensation mechanisms. The predominance of standing rumination during NT in the rainy season suggests post-grazing digestive activity concentrated in cooler conditions, consistent with Serranito et al. (51) and Martin et al. (52). During the less rainy season, the higher occurrence of standing rumination in MH indicates that animals adjust rumination timing to periods of moderate thermal load, favoring gastrointestinal efficiency (53–55). In TS, the concentration of rumination in daylight shifts may reflect limited resting infrastructure or greater exposure (56–59).

Lying rumination exhibited strong seasonal modulation, with greater expression during thermally favorable shifts, supporting the interpretation that recumbent rumination is associated with comfort conditions (60–65). However, differences between SP and TS demonstrate that system structure influences how animals distribute rumination spatially and temporally, reinforcing the importance of microclimatic stability (66–69).

Overall, the interaction between seasonality and production system was not merely additive but synergistic. The silvopastoral system consistently promoted greater behavioral balance across shifts, particularly during the rainy season, while the traditional system showed more pronounced temporal concentration of activities, especially during cooler hours (71–79). These findings are consistent with the literature, suggesting that structural environmental complexity enhances welfare, yet our results add nuance by demonstrating that mitigation effects vary according to season and time of the day (70).

## Data Availability

The datasets presented in this study can be found in online repositories. The names of the repository/repositories and accession number(s) can be found in the article/supplementary material.
